# High-efficiency electrochemical thermal energy harvester using carbon nanotube aerogel sheet electrodes

**DOI:** 10.1038/ncomms10600

**Published:** 2016-02-03

**Authors:** Hyeongwook Im, Taewoo Kim, Hyelynn Song, Jongho Choi, Jae Sung Park, Raquel Ovalle-Robles, Hee Doo Yang, Kenneth D. Kihm, Ray H. Baughman, Hong H. Lee, Tae June Kang, Yong Hyup Kim

**Affiliations:** 1 School of Mechanical and Aerospace Engineering, Seoul National University, Seoul 151-742, South Korea; 2 Institute of Advanced Machinery and Design, Seoul National University, Seoul 151-742, South Korea; 3 Nano-Science & Technology Center, Lintec of America, Inc., Richardson, Texas 75081, USA; 4 Department of NanoMechatronics Engineering, College of Nanoscience and Nanotechnology, Pusan National University, Busan 609-735, South Korea; 5 Department of Mechanical, Aerospace and Biomedical Engineering, University of Tennessee, Knoxville, Tennessee 37996, USA; 6 Alan G. MacDiarmid NanoTech Institute, University of Texas at Dallas, Richardson, Texas 75080, USA; 7 School of Chemical and Biological Engineering, Seoul National University, Seoul 151-744, South Korea; 8 Department of Mechanical Engineering, INHA University, Incheon 22212, South Korea; 9 Institute of Advanced Aerospace Technology, Seoul National University, Seoul 151-742, South Korea

## Abstract

Conversion of low-grade waste heat into electricity is an important energy harvesting strategy. However, abundant heat from these low-grade thermal streams cannot be harvested readily because of the absence of efficient, inexpensive devices that can convert the waste heat into electricity. Here we fabricate carbon nanotube aerogel-based thermo-electrochemical cells, which are potentially low-cost and relatively high-efficiency materials for this application. When normalized to the cell cross-sectional area, a maximum power output of 6.6 W m^−2^ is obtained for a 51 °C inter-electrode temperature difference, with a Carnot-relative efficiency of 3.95%. The importance of electrode purity, engineered porosity and catalytic surfaces in enhancing the thermocell performance is demonstrated.

## Abstract (web summary)

Conversion of low-grade waste heat into electricity is an important energy harvesting strategy. Here, the authors fabricate carbon nanotube aerogel-based thermo-electrochemical cells, which are potentially low-cost and relatively high-efficiency materials for this application.

## Graphical abstract

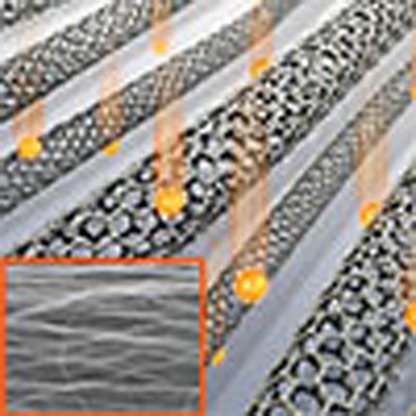

Harvesting energy from waste heat has received much attention due to the world's growing energy problem^1^,^2^,^3^,^4^. Critical needs for harnessing waste heat are to improve the efficiency of thermal energy harvesters and decrease their cost^5^. Solid-state thermoelectric devices have been long investigated for the direct conversion of thermal energy to electrical energy, and many exciting advances have been made^6^,^7^. However, device performance relative to cost has so far limited application for waste heat recovery^8^. Thermal electrochemical energy harvesters^9^ might have major advantages, as suggested in a very preliminary way by previous comparisons of Wh/dollar of solar and electrochemical thermocells^10^; however, they presently have no commercial applications because of their low energy conversion efficiencies and low areal output power. The goal of the present work is to increase the obtained energy conversion efficiencies and areal output power of thermocells to the point where they outperform thermoelectrics in energy output per device cost during device lifetime for low-grade thermal energy harvesting.

To increase the energy conversion efficiency of thermocells, carbon nanomaterials have been introduced as cell electrodes to take advantage of the fast redox processes, high thermal and electrical conductivities, and high gravimetric surface areas that these materials can provide^11^. Hu *et al.*^10^ reported an energy conversion efficiency as high as 1.4%, relative to Carnot cycle efficiency, when carbon multi-walled nanotube (MWNT) buckypaper was used for thermocell electrodes. This efficiency was raised to 2.6% by introducing a carbon single-walled nanotube (SWNT)/reduced graphene oxide (rGO) composite electrode^12^. Improved mass transport due to enhanced porosity of the optimized SWNT/rGO composite was found responsible for the efficiency enhancement. Despite recent advances in thermocell technology, a significant efficiency increase is required for thermocells to become commercially attractive, considering that the Carnot-relative efficiency has to be 2–5% for commercial viability^13^. While efficiency is undoubtedly a key factor, another important quantity is the specific power the device can generate.

Here we exploit planar and cylindrically wound carbon nanotube (CNT) aerogel sheets as thermocell electrodes and devise various additional ways to optimize Carnot efficiency. The deployed optimization strategies to improve thermocell performance involve the use of CNT aerogel sheets as electrodes, removal of low activity carbonaceous impurities that limit electron transfer kinetics, decoration of CNT sheets with catalytic platinum nanoparticles, mechanical compression of nanotube sheets to tune conductivity and porosity, and the utilization of a cylindrical cell geometry. The output power density generated by a described cylindrical thermocell reaches 6.6 W m^−2^ for a 51 °C inter-electrode temperature difference, which corresponds to a Carnot-relative efficiency of 3.95% (that is, 3.95% of the maximum energy conversion efficiency possible for a heat engine operating between two given temperatures).

## Results

### CNT aerogel sheets as high-performance electrodes

**Figure 1:**
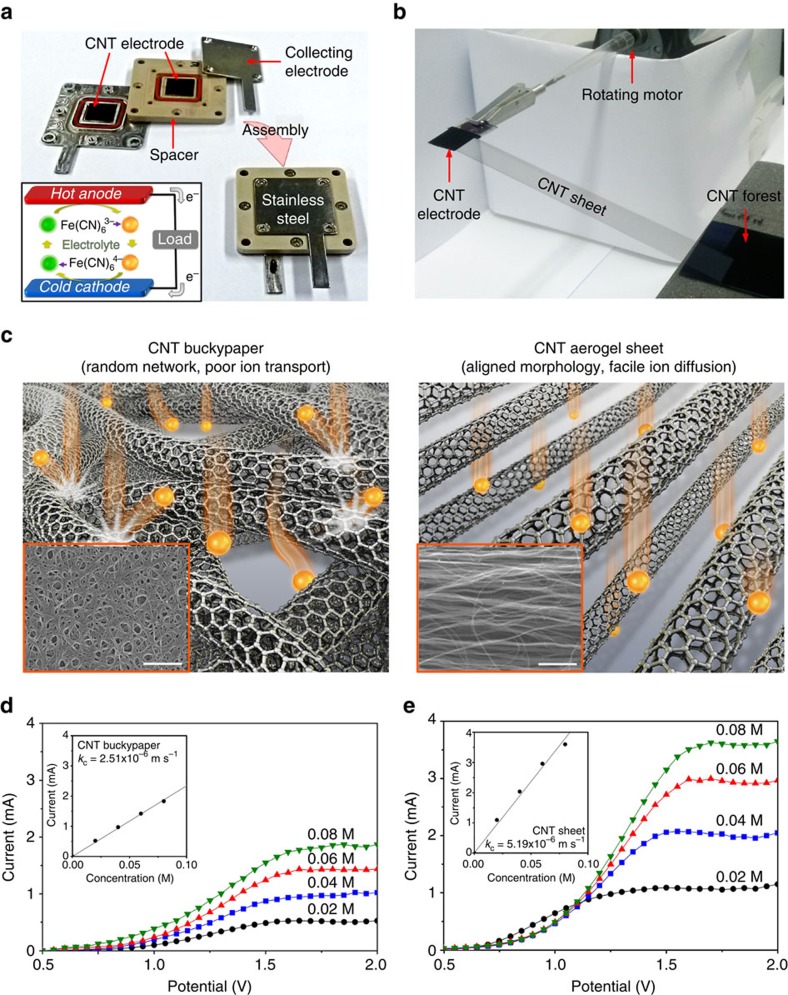
Carbon nanotube aerogel sheets as high-performance electrodes. (**a**) Photographs of cell components and their assembly into a planar thermocell (inset: schematic drawing of thermocell operation). (**b**) An apparatus for continuously drawing a carbon nanotube (CNT) aerogel sheet from a CNT forest, and wrapping it around a metal frame to form an electrode for a planar thermocell. (**c**) Illustrations and SEM micrographs (insets) comparing CNT buckypaper and CNT aerogel electrodes and the relationship of these morphologies to ion transport (scale bars in the insets, 1-μm). MWNT bundling is not shown and only MWNT outer walls are pictured. Polarization curves for (**d**) CNT buckypaper and (**e**) CNT aerogel electrodes. The insets show the dependence of limiting current on ferrocyanide concentration.

A generic aqueous electrolyte thermocell, which utilizes the Fe(CN)_6_^4−^/Fe(CN)_6_^3−^ redox couple and K^+^ as the counter ions, is schematically illustrated in the inset of Fig. 1a. An inter-electrode temperature difference causes a difference in the redox potentials of the electrolyte at the electrodes. This thermally generated potential difference drives electrons in the external circuit and ions in the electrolyte, thereby enabling electrical power to be generated. Continuous operation of the thermocell requires transport of the reaction products formed at one electrode to the other electrode. If either electrode is not furnished with the redox molecules needed for electron generation or consumption, then power production will cease.

The thermoelectric potential in a thermocell is generated by the temperature dependence of the free energy difference between reactant and product of a reaction taking place at the electrolyte–electrode interface^9^,^13^. Minor effects on the cell potential arise from the thermal diffusion (Soret effect)^13^,^14^,^15^,^16^ and the transport entropy of ions^17^. However, for most systems of interest, the electrode potentials dominate and the minor effects on potential can be neglected for practical purposes^13^,^16^.

A planar-type thermocell, shown in Fig. 1a, was used to investigate the potential of CNT aerogel sheets as high-performance electrodes. For preparation of the thermocell electrode, CNT aerogel sheet was drawn from a CNT forest and laid on a rectangular tungsten frame that is connected to a motor. Then, the motor was rotated at 10 r.p.m. to simultaneously draw the sheet from the forest and warp it onto the frame (Fig. 1b). The thickness and area of the CNT electrode can be easily controlled by the number of motor rotations and the frame size, respectively. A 100-μm-thick CNT sheet electrode with an area of 1.0 × 1.0 cm^2^ was used to evaluate thermocell performance. All thermocells and all electrochemical impedance measurements (to determine equivalent series resistance (ESR) and charge transfer resistance (*R*_ct_)) used as electrolyte a 0.4 M aqueous solution of potassium ferro/ferricyanide. Cyclic voltammetry (CV) measurements used an aqueous solution of 10 mM K_3_Fe(CN)_6_ and 0.1 M KCl.

Fast transport of the redox mediator ions into electrodes is required to obtain high-areal power generation from CNT thermocells. Randomly oriented CNT sheets, such as CNT buckypaper, may impede ion transport into electrode depths, due to the high tortuosity of the pore structure. On the other hand, the well-aligned CNTs in the aerogel sheets might result in faster ion transport deep within the electrodes, as schematically illustrated in Fig. 1c. The effectiveness of ion transport in CNT aerogel sheets was characterized by measuring the mass transport coefficient using an electrolytic flow cell (see Methods and Supplementary Fig. 1). Exploiting the limiting-current method, the mass transfer coefficient (*k*_c_) was estimated from the dependence of limiting current on electrolyte concentration by using the following equation^18^,^19^:


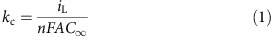


where *i*_L_ is the limiting current, *n* is the number of moles of electrons transferred, *F* is the faradaic constant, *A* is the electrode area and *C*_∞_ is the bulk species concentration.

Various aqueous 1:2 concentrations of Fe(CN)_6_^3−^/Fe(CN)_6_^4−^ redox couple in 0.5 M NaOH were used for the experiments, by varying the Fe(CN)_6_^3−^ concentration from 20 to 80 mM. The concentration of Fe(CN)_6_^4−^ was twice the Fe(CN)_6_^3−^ concentration for each experiment to ensure a limiting reaction rate at the cell cathode (that is, Fe(CN)_6_^3−^+e^−^→Fe(CN)_6_^4−^). The electrolyte stored in a glass container was circulated through the cell by a peristaltic pump. The flow rate was kept low at 6.6 × 10^−6^ m^3^ s^−1^ for allowing laminar flow (Reynolds number, Re∼650, Supplementary Note 1 and Supplementary Table 1) in the cell.

The limiting-current method is based on driving an electrochemical reaction to the maximum possible reaction rate, which is limited by the mass transport of redox ions. The reaction rate limit is indicated by a current plateau on a polarization curve plot. Polarization curves of the thermocells based on CNT buckypaper and CNT aerogel sheet electrodes are shown in Fig. 1d,e, respectively. A linear relationship between limiting current and reactant concentration is evident from the insets of the figure panels. Using equation (1), the mass transfer coefficient of CNT aerogel sheet (5.19 × 10^−6^ m s^−1^) is estimated to be twice that of CNT buckypaper (2.51 × 10^−6^ m s^−1^). The transfer coefficient of CNT aerogel sheet electrode approaches the theoretically limiting mass transfer coefficient (5.76 × 10^−6^ m s^−1^, see Supplementary Note 1), which corresponds to the unobstructed transport of reactants to a flat plate. These electrochemical results and the SEM micrographs suggest that the low tortuosity of the pore structure in the CNT aerogel, compared with that of CNT buckypaper, results in faster ion diffusion to deep within the electrode and corresponding higher limiting currents at a given redox concentration.

### Optimization of thermocell performance

It is well-known that impurities, such as carbonaceous byproducts, are introduced during CNT synthesis^20^. A coating of these impurities on CNTs could restrict charge exchange with redox ions in the electrolyte, thereby degrading cell performance^21^. Various efforts have been made to remove undesirable amorphous carbon from CNT surfaces, for the purpose of improving performance for diverse applications of CNTs^21^,^22^. We take advantage of the difference in oxidation rate in air between CNT and byproducts^23^ to purify forest-drawn CNT sheets, which are ultimately used as electrodes.

**Figure 2:**
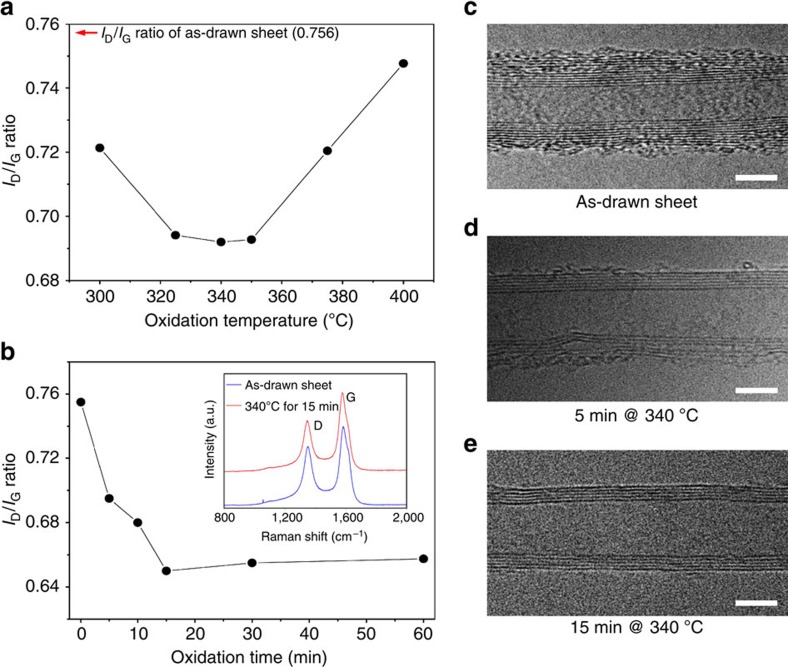
The effects of thermal oxidation temperature and time. (**a**) The dependence of Raman *I*_D_/*I*_G_ ratio on oxidation temperature for a 5-min anneal of CNT aerogel sheets in ambient air. (**b**) The dependence of the *I*_D_/*I*_G_ ratio for CNT aerogel sheets on oxidation time in ambient air at 340 °C. The inset shows the Raman spectra for an as-drawn CNT sheet and for a CNT sheet that has been thermally oxidized in air for 15 min at 340 °C. (**c**–**e**) High-resolution transmission electron microscope (HR-TEM) images of (**c**) as-drawn, (**d**) 5-min oxidized, and (**e**) 15-min oxidized CNT aerogel sheets (all scale bars, 5-nm).

To find the optimum heating schedule, the oxidation temperature was first varied in the range of 300–400 °C, while the oxidation time was held constant at 5 min. The purity of the CNT sheets was characterized using Raman spectroscopy for 514 nm excitation. The effect of oxidation temperature on the Raman intensity ratio of D band to G band (*I*_D_/*I*_G_) is shown in Fig. 2a, where the D band peak corresponds to defective carbon (like in amorphous carbon or carbon in defect sites) and the G band arises from ordered sp^2^ carbons^24^. The minimum ratio *I*_D_/*I*_G_ occurs at anneal temperatures between 325 and 350 °C, signifying that higher anneal temperatures should not be used for annealing times as short as 5 min. For further optimization of CNT aerogel sheet quality, the anneal temperature was fixed at 340 °C and the heating time was varied between 0 and 60 min to further minimize the *I*_D_/*I*_G_ ratio (Fig. 2b). The Raman spectra for an as-drawn sheet and for the optimally annealed sheet are shown in the figure inset. The merit of this method for removing CNT impurities was confirmed by high-resolution transmission electron microscopy (HR-TEM) images of MWNTs in the annealed sheets. The non-annealed MWNT (Fig. 2c) is covered with a carbonaceous coating, which is reduced by the 5-min anneal at 340 °C (Fig. 2d), and eliminated by a 15-min anneal at 340 °C (Fig. 2e), which corresponds to the minimum obtained ratio of *I*_D_/*I*_G_ in Fig. 2b.

**Figure 3:**
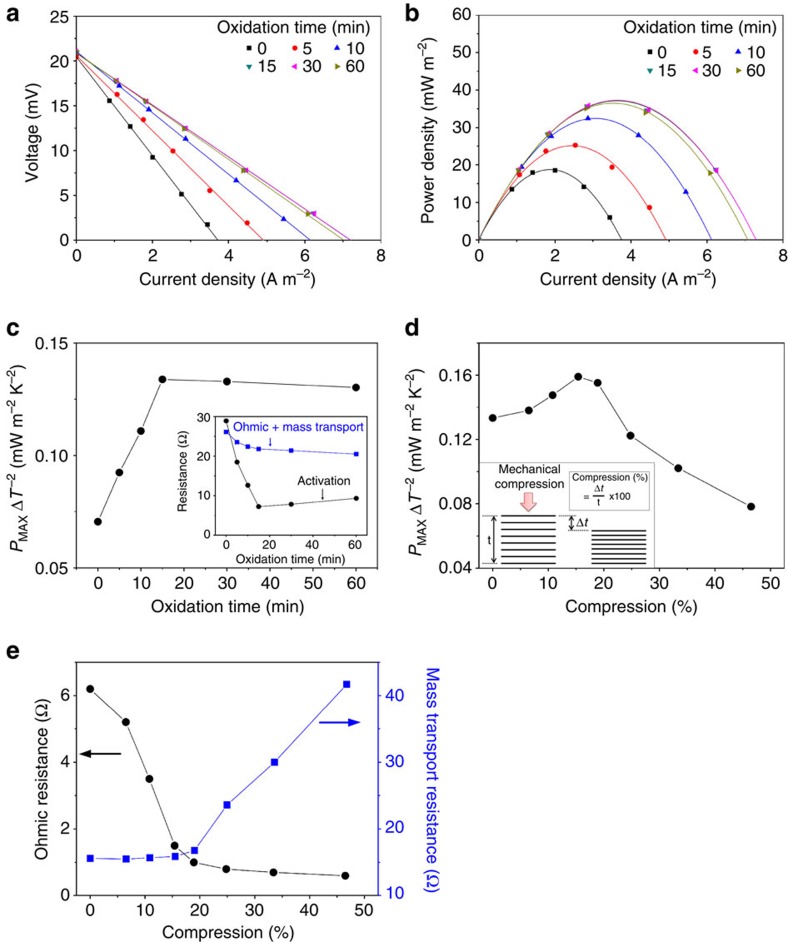
The effect on thermocell performance of oxidation and compression of the CNT aerogel. (**a**) Cell voltage versus current density and (**b**) cell power density versus current density for samples having different thermal oxidation times. (**c**) Maximum power density normalized to the inter-electrode temperature difference (*P*_MAX_/Δ*T*^2^) as a function of thermal oxidation time. (Inset: the dependence of activation resistance and the sum of ohmic and mass transport resistance on thermal oxidation time). (**d**) *P*_MAX_/Δ*T*^2^ generated by the thermocell as a function of the per cent mechanical compression of cell electrodes. The inset illustrates the mechanical compression of a planar CNT aerogel electrode. (**e**) The dependence of ohmic and mass transport resistance on the compressive strain shown in **d**.

Thermocell performance was strongly affected by the annealing time at 340 °C, as shown in Fig. 3a,b. In this experiment, the hot plate temperature was maintained at 25 °C and the cold plate temperature was at 5 °C. While this temperature difference (Δ*T*) of 20 °C was applied between heating and cooling plates, the actual Δ*T* between the electrodes, which was calculated using the open-circuit voltage and the observed thermo-electrochemical Seebeck coefficient (1.43 mV K^−1^)^16^, was smaller (17.5 °C). This difference is caused by thermal resistances (and corresponding temperature drops) at the interfaces between hot and cold electrodes and the respective heating and cooling plates. The curves of cell voltage versus areal current density and the corresponding power density curves obtained for various thermal oxidation times (shown in Fig. 3a,b) reveal that a lower *I*_D_/*I*_G_ ratio, indicating that a cleaner CNT sheet, yields a higher maximum output power. The maximized areal power density (*P*_MAX_), normalized to the square of the inter-electrode temperature difference (Δ*T*^2^), is shown as a function of thermal oxidation time at 340 °C in Fig. 3c. A 186% increase (from 0.07 to 0.13 mW m^−2^ K^−2^) in the *P*_MAX_/Δ*T*^2^ is shown to result from removing carbonaceous impurities from the surfaces of CNTs and CNT bundles. This optimum thermal oxidation condition (15 min at 340 °C) was used for further experiments.

To explain this major improvement in performance, we used electrochemical impedance measurements to characterize the three primary internal resistances of the thermocell (that is, the activation, ohmic and mass transport resistances)^16^,^25^. The activation resistance is the loss incurred in overcoming the activation barrier associated with reactions at the electrodes. The ohmic resistance is mainly due to the series resistances of electrode and electrolyte and the mass transport resistance is associated with the kinetics of ion diffusion and convection in the thermocell. The changes in these internal resistances with respect to heating time during sheet oxidation at 340 °C are shown in the inset of Fig. 3c. This figure shows that a major reduction in the activation resistance is realized by removal of carbonaceous impurities, while the decrease in the combined ohmic and mass transport resistances is much smaller. This result indicates that amorphous carbon covering the surface of CNT hinders electrochemical reaction, resulting in performance degradation.

The fact that the electrode is an aerogel implies that increased contact between CNTs (and correspondingly decreased inter-electrode electrical and thermal resistances) could be realized by compressing the electrode. However, the correspondingly increased density can restrict ion transport into the interior of the electrode (see Supplementary Fig. 2). To evaluate these opposing effects, mechanical compression of a planar electrode was accomplished by pressing CNT sheets (wrapped on the tungsten frame used for electrode fabrication), using two silicon wafers that were coated with gold films (which provide low adhesion to the CNT sheet stack). The maximum obtained power density, normalized to Δ*T*^2^, is plotted in Fig. 3d as a function of the per cent compression in the sheet thickness direction. These results show that *P*_MAX_/Δ*T*^2^ is maximized for ∼15% sheet compression, and that this compression provides a 23% increase in this performance metric (from 0.13 to 0.16 mW m^−2^ K^−2^).

The data in Fig. 3e show the origin of this effect of sheet compression. The mass transport resistance increases with increasing level of compression, whereas the ohmic resistance decreases with increasing compression. Ohmic resistance decreases with increasing compression, because a shorter electron pathway to the external circuit results from an increased number of contacts between CNTs and a decreased electrode thickness. On the other hand, mass transport resistance, typically affected by ion accessibility into electrodes, is increased by compression, indicating sluggish ion diffusion within the compressed CNT network (Supplementary Fig. 2). Therefore, *P*_MAX_/Δ*T*^2^ is maximized at an intermediate compression (15%), where mass transport resistance is not much affected by compression, but ohmic resistance is dramatically decreased.

**Figure 4:**
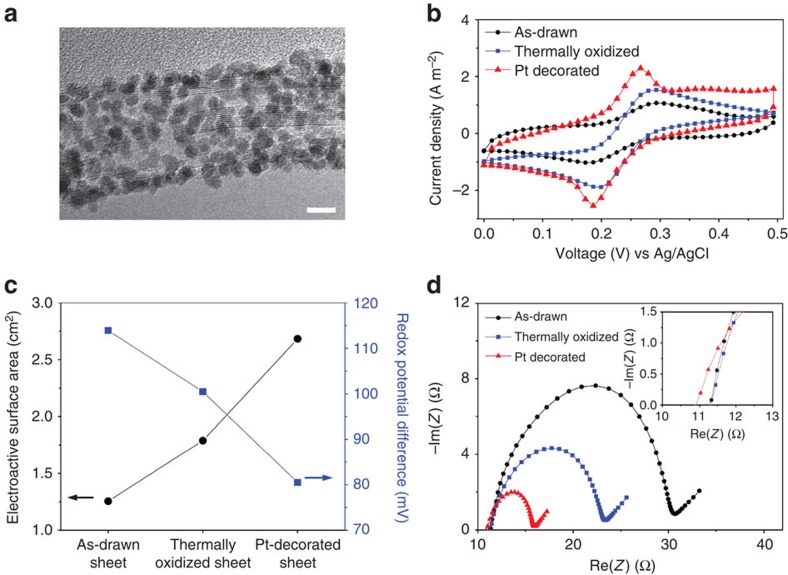
Pt nanoparticle deposition for improving electrode performance. (**a**) HR-TEM image of individual CNT decorated by Pt nanoparticles. Scale bar, 5 nm. (**b**) Cyclic voltammograms obtained at a 100 mV s^−1^ scan rate using as-drawn, thermally oxidized, and thermally oxidized and Pt-deposited CNT electrodes. (**c**) Electroactive surface area and redox potential difference for the above various CNT electrodes. (**d**) Nyquist impedance plots for various electrodes. The inset shows a close-up of the high frequency region of the curves.

To further improve electrode performance, Pt nanoparticles were deposited on the thermally oxidized CNT aerogel sheet electrodes by chemical reduction of a platinum salt solution (see Methods and Supplementary Fig. 3). This Pt nanoparticle deposition increases the electrode surface area for the thermocell reaction, and the catalytic activity of platinum reduces the charge transfer resistance^26^. The HR-TEM image of Fig. 4a and the statistical size analysis of deposited Pt nanoparticles in Supplementary Fig. 3b show that platinum nanoparticles with an average diameter of 2.4 nm are uniformly deposited on the surfaces of individual CNTs and nanotube bundles to provide 86 wt% of Pt in the electrodes.

CV measurements were used to obtain the changes in electroactive surface area (ESA)^27^ and redox potential difference between oxidation and reduction that Pt deposition provided. The CV curves of Fig. 4b indicate an increase in the Faradaic peak current for the thermally oxidized sheet electrode and Pt-decorated thermally oxidized sheet electrode (Pt-sheet) compared with that for the as-drawn sheet electrode. Moreover, the Pt-sheet has the highest faradaic current. This increased current can be attributed to the increased ESA, which according to the Randles–Sevcik equation^28^ is given by:


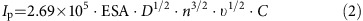


where *I*_p_ is the faradaic peak current, *D* is the diffusion coefficient, *n* is the number of electrons transferred during the redox reaction, *υ* is the potential scan rate, and *C* is the concentration of probe molecule.

The redox potential differences and ESAs determined by CV are shown in Fig. 4c for electrodes that are as-drawn, thermally oxidized and platinum-deposited after chemical cleaning by thermal oxidation. This figure shows that the cleaned CNT electrode has a higher ESA and a smaller redox potential difference than the as-drawn CNT electrode, which led to the improved performance shown in Fig. 3c. The largest ESA and the lowest potential difference resulted (Fig. 4c) when Pt nanoparticles were deposited onto the cleaned surface of CNTs. These results indicate that the Pt-decorated sheet electrodes provide the highest performance in thermocells, because they yield the largest effective surface area and the greatest reduction in the potential difference between peaks for oxidation and reduction. Electrochemical impedance spectroscopy analysis was also performed to support the performance improvement. The ESR (the intercept of the curve with the *x*-axis of the Nyquist plot^29^ in the inset of Fig. 4d) is slightly reduced after Pt decoration. The Nyquist plot in Fig. 4d shows that the charge transfer resistance (the diameter of the semicircle)^30^ is lower for the thermally oxidized sheet than for the as-drawn sheet and that it is lowest for the Pt-decorated, thermally oxidized sheet. These observations from electrochemical impedance spectroscopy measurements agree with the CV results.

### Cylinder-type CNT thermocells and their performance

**Figure 5:**
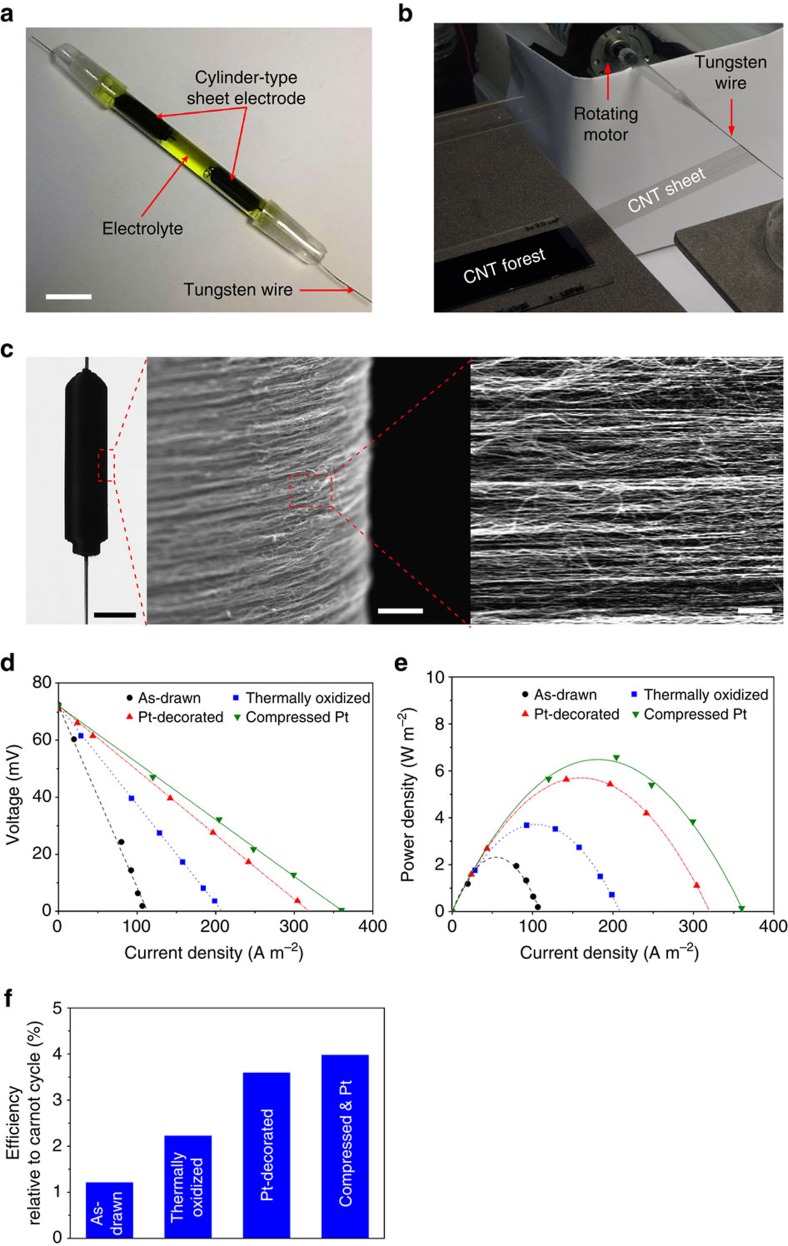
Fabrication of cylinder-type CNT thermocells and their performance for Δ*T*≈51 °C. (**a**) Photograph of an assembled thermocell (scale bar, 2 cm) and (**b**) apparatus for fabrication of cylindrical CNT thermocell electrodes. (**c**) Photograph and SEM images of a cylindrical CNT electrode. Scale bars, 3 mm (left of panel), 30 μm (middle of panel) and 1 μm (right of panel). (**d**) Cell voltage and (**e**) power density versus current density for variously treated cell electrodes. (**f**) Energy conversion efficiency relative to Carnot cycle efficiency for various CNT electrodes in the cylindrical cell configuration. All sheet samples (except the as-drawn) were purified by thermal oxidation before assembly into cylindrical electrodes.

Building on our results for planar electrodes, we further optimized thermocell performance by using the cylindrical electrode configuration of Fig. 5a (while deploying the same 0.4 M ferro/ferricyanide aqueous electrolyte as for the flat thermocells). The cylindrical electrodes pictured in this figure were fabricated by first using the apparatus of Fig. 5b to wind a forest-drawn CNT sheet onto a 300-μm diameter tungsten wire (which facilitates current collection) until a 3.0- to 3.5-mm outer scroll diameter was obtained. The SEM images of Fig. 5c show the realized highly uniform structure of the electrode sidewall and the orientation of the CNTs around the electrode circumference. Using the results of Fig. 3d on the optimal degree of electrode compression to produce densification, a cleaned CNT sheet electrode (wound to a 3.5 mm diameter) was compressed to 3.0 mm diameter by using two grooved templates (shown in Supplementary Fig. 4). Following deposition of Pt nanoparticles by chemical reduction of a platinum salt solution, two resulting cylindrical CNT sheet electrodes were inserted into a 3 mm inner diameter, cylindrical glass tube to form the thermocell of Fig. 5a.

The performance of the above thermocells containing differently prepared cylindrical CNT aerogel electrodes is presented in Fig. 5d,e for Δ*T*≈51 °C. The cylindrically configured as-drawn sheet electrode generates a *P*_MAX_ of 2.0 W m^−2^. These results show that removal of carbonaceous impurities by the described optimized thermal oxidation increases the power output to 3.7 W m^−2^, which is further increased for the cylindrical electrode thermocell to 6.0 W m^−2^ when Pt nanoparticles are deposited within the cylindrical electrodes. Finally, the maximum power density was further enhanced to 6.6 W m^−2^ when the cleaned, Pt-sheet electrode was laterally compressed. This output corresponds to an energy conversion efficiency of 3.95%, relative to the theoretically limiting Carnot cycle efficiency, as shown in Fig. 5f.

The energy conversion efficiency (*η*) of the thermocells was calculated using


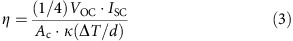


where *V*_OC_ is the open-circuit voltage, *I*_SC_ is the short-circuit current, *A*_c_ is the cross-sectional area of the cell, *κ* is the thermal conductivity of the electrolyte, Δ*T* is the temperature difference between the electrodes and *d* is the electrode separation distance (see Supplementary Note 2).

The open-circuit voltage was *V*_OC_=72 mV and the Δ*T* thereby calculated (from this voltage and the Seebeck coefficient of 1.43 mV K^−1^, Supplementary Note 3, and Supplementary Fig. 5b) was 51.4 °C for all electrode types in the cylindrical thermocell configuration. Thermal conduction dominates the heat transfer through the electrolyte between the hot and cold electrodes (see Supplementary Note 4, Supplementary Fig. 6 and Supplementary Table 2). The cell area and the thermal conductivity^31^ are 7.1 × 10^−6^ m^2^ and 0.57 W m^−1^ K^−1^, respectively. The electrode separation distance is 2.5 cm, corresponding to the closest approach distance between the hot and cold electrodes shown in Fig. 5a. The short-circuit current obtained from as-drawn, thermally oxidized, thermally oxidized and Pt-decorated; and thermally oxidized, compressed and Pt-decorated sheets in the cylindrical cell configuration are 0.8 mA, 1.5 mA, 2.3 mA and 2.6 mA, respectively. Using equation (3), *η* values of 0.17%, 0.32%, 0.51% and 0.56% are attained through the use of cylindrical thermocell configuration for these cylindrical thermocells from differently processed CNT aerogel sheets, respectively.

The energy conversion efficiency (*η*_r_), relative to the Carnot efficiency limit of a heat engine, is


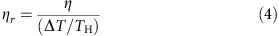


where *T*_H_ is the hot temperature. We calculated the Carnot-relative energy conversion efficiency using equations (3) and (4) (See Supplementary Table 3 for parameters used in the equations), the measured Δ*T*=51.4 °C, and an estimated *T*_H_ for the hot side electrode temperature of 90.7 °C (estimated by considering symmetric geometry of cylindrical cell and electrodes arrangement, and Δ*T*). The resulting calculated Carnot-relative efficiency was *η*_r_=3.95% for the optimized cylindrical thermocell (based on cylindrical electrodes made of thermally purified, optimally compressed, Pt-decorated CNT sheets). This thermocell efficiency is substantially higher than the previously reported record Carnot-relative efficiency for a thermocell, which is 2.63% (ref. 12).

## Discussion

The ability to simultaneously obtain a record Carnot-relative efficiency (3.95%) and a record areal current density (6.6 W m^−2^ for a temperature difference of only 51.4 °C) bodes well for eventual practical deployment of thermo-electrochemical cells for harvesting low-grade heat as electrical energy. This work demonstrates the importance of electrode purity, engineered porosity and catalytic surfaces on thermocell performance and the ways that each predominately affects thermocell parameters. It is doubtful that thermal electrochemical cells will ever match the efficiencies obtained for costly thermoelectrics. However, potentially low cost (using future low-cost catalyst) and convenient deployability (like the demonstrated wrapping of flexible CNT sheet thermocells about hot pipes^10^) might eventually lead to their importance in the menagerie of means for practically harvesting thermal energy.

## Methods

### Mass transfer coefficients of CNT electrodes

The mass transport characteristics of CNT buckypaper and CNT aerogel sheet electrodes were investigated using the electrolytic flow cell of Supplementary Fig. 1. CNT buckypaper was prepared by vacuum filtration of MWNTs (CM-95, Hanwha Nanotech) suspended in anhydrous N,N-dimethylformamide (N,N-DMF, Sigma-Aldrich) onto a membrane filter (Millipore PTFE filter, 0.2 μm pore size, 47 mm diameter), washing with deionized (DI) water and methanol, drying in vacuum and removal of the formed sheet from the filter. CNT aerogel sheet electrodes were prepared by sheet draw from a CNT forest. The electrolytic flow cell shown in Supplementary Fig. 1 consists of two parallel collecting electrodes that are separated by 10 mm. For both the CNT buckypaper and CNT aerogel sheet evaluations, 100-μm-thick CNT electrodes having an identical area of 1.0 × 1.0 cm^2^ are attached on the centre of both collecting electrodes using carbon paste. Other exposed areas of the collecting electrodes were covered with 100-μm-thick polyethylene terephthalate film. Various aqueous 1:2 concentrations of Fe(CN)_6_^3−^/Fe(CN)_6_^4−^ redox couple in 0.5 M NaOH were used for the experiments by varying the Fe(CN)_6_^3−^ concentration from 20 to 80 mM. The concentration of Fe(CN)_6_^4−^ was twice as high as the Fe(CN)_6_^3−^ concentration for each experiment to ensure a limiting reaction rate at the cell cathode (that is, Fe(CN)_6_^3−^+e^−^→Fe(CN)_6_^4−^). The electrolyte stored in a glass container was circulated through the cell by a peristaltic pump (Longer pump, BT100-2J), and the flow rate was kept low at 6.6 × 10^−6^ m^3^ s^−1^ for allowing laminar flow in a cell channel. A power supply (Keithley, 2400 Sourcemeter) was used to drive electrochemical reactions.

### Preparation and post-treatment of CNT aerogel sheet

Vertically aligned MWNT arrays were grown on an iron-catalyst-coated silicon (Si) substrate by chemical vapour deposition of acetylene gas^32^. The diameter and height of MWNTs are ∼10 nm and ∼200 μm, respectively. CNT sheet was continuously drawn from a sidewall of the MWNT forests using a dry spinning process^32^,^33^ and wrapped around a rectangular tungsten frame for planar-type electrodes and tungsten wire for cylinder-type electrodes, using a connected rotating motor at 10 r.p.m. The planar electrode has a thickness of 100 μm and area of 1.0 × 1.0 cm^2^ and the cylinder electrodes had a diameter and length of 3 mm and 2.0 cm, respectively. Thermal annealing of the CNT sheets to remove carbonaceous impurities was performed in ambient atmosphere using a halogen-lamp-heated quartz tube furnace, which had a ramp time to target temperatures (from 300 to 400 °C) of 1 min. For deposition of Pt nanoparticles by chemical reduction of a platinum salt solution^34^, 3.75 mg of K_2_PtCl_4_ (Aldrich) was dissolved in 50 ml of diluted ethylene glycol solution (3:2 by volume ethylene glycol:DI water). Afterwards, the described thermally oxidized CNT sheet electrodes were immersed in the platinum salt solution and Pt nanoparticle deposition was permitted for 3 h at 110 °C with weak stirring to prevent mechanical damage to CNT sheet. After the reaction, the pH of the solution was decreased to 2 using HCl and the electrode was washed with DI water several times to remove excess ethylene glycol. To avoid liquid-based aerogel densification during liquid evaporation, the electrodes were not dried before use in the thermocells.

### Thermocell testing and materials characterization

The electrolyte used in all tests was a 0.4 M aqueous solution of Fe(CN)_6_^4−^/Fe(CN)_6_^3−^. The ionic conductivity of the electrolyte at different temperatures was measured using a conductivity meter (Metter Toledo S-230), as shown in Supplementary Fig. 7. For evaluation of planar thermocells, the cell (Fig. 1a) was placed between two fluid-heated plates that were connected to hot and cold thermostatic baths to provide ±0.1 °C control of plate temperatures. For cylindrical thermocell testing, the glass wall surrounding each cell electrode (which are separated by 2.5 cm) was wrapped and bonded with a TYGON tube, through which cooling fluid or heating fluid was passed. The inter-electrode direction of the thermocell was oriented horizontally during measurements. While the hot and cold fluid temperatures were 100 °C and 30 °C, respectively, thermal resistances between the heating and cooling sources and the respective electrodes reduced the inter-electrode temperature difference from 70 °C to Δ*T*=51.4 °C. This Δ*T* of 51.4 °C, obtained by dividing the measured *V*_OC_ by the thermo-electrochemical Seebeck coefficient, would correspond to a temperature difference between the cold and hot electrodes averaged over the radial shape of CNT electrode. A voltage-current meter (Keithley, 2000 Multimeter) was used for characterizing cell voltage versus cell current for different external resistive loads, and thereby determining power output. The glass tube having 3 mm inside diameter was used for the cylinder thermocell experiments (Fig. 5a). Raman spectra as a function of the thermal oxidation times and the temperature used for removing carbonaceous impurities were recorded for 514 nm excitation using a Renishaw inVia Raman Microscope. Sample structure was further characterized by field-emission scanning electron microscopy (FE-SEM, Hitachi-S4800) and transmission electron microscopy (JEM-300F). Electrochemical impedance measurements were conducted in the frequency range between 10 kHz and 50 mHz using a commercial instrument (Zahner, IM6ex). CV (using a Digi-Ivy, DY2100 instrument) used 10 mM K_3_Fe(CN)_6_ with 0.1 M KCl as the supporting electrolyte in aqueous solution and a scan rate of 100 mV s^−1^. Platinum and Ag/AgCl electrodes were used as counter and reference electrodes, respectively, for the CV measurements.

## Additional information

**How to cite this article**: Im, H. *et al.* High-efficiency electrochemical thermal energy harvester using carbon nanotube aerogel sheet electrodes. *Nat. Commun.* 7:10600 doi: 10.1038/ncomms10600 (2016).

## Supplementary Material

Supplementary Information Supplementary Figures 1-7, Supplementary Tables 1-3, Supplementary Notes 1-4 and Supplementary References
