# Supplementary material for: High-efficiency electrochemical thermal energy harvester using carbon nanotube aerogel sheet electrodes
Source: Nat Commun. 2016 Feb 3;7:10600. doi: 10.1038/ncomms10600 (PMC4742963; doi:10.1038/ncomms10600)
Supplement: Supplementary Information — Supplementary Figures 1-7, Supplementary Tables 1-3, Supplementary Notes 1-4 and Supplementary References [file ncomms10600-s1.pdf]

Supplementary Figures

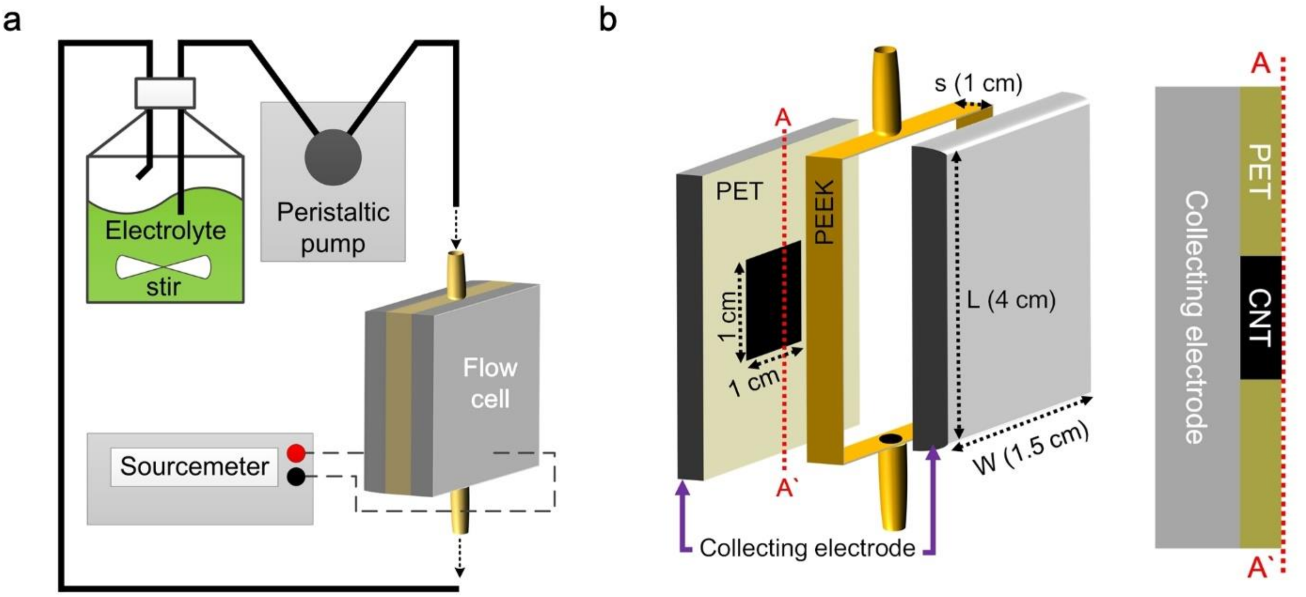

Supplementary Figure 1. Characterization of the effectiveness of ion transport in CNT aerogel sheets. (a) Schematic drawing of experimental setup for measuring mass transfer coefficient. (b) Exploded view of the flow cell. PET is polyethylene terephthalate and PEEK is polyester ether ketone.

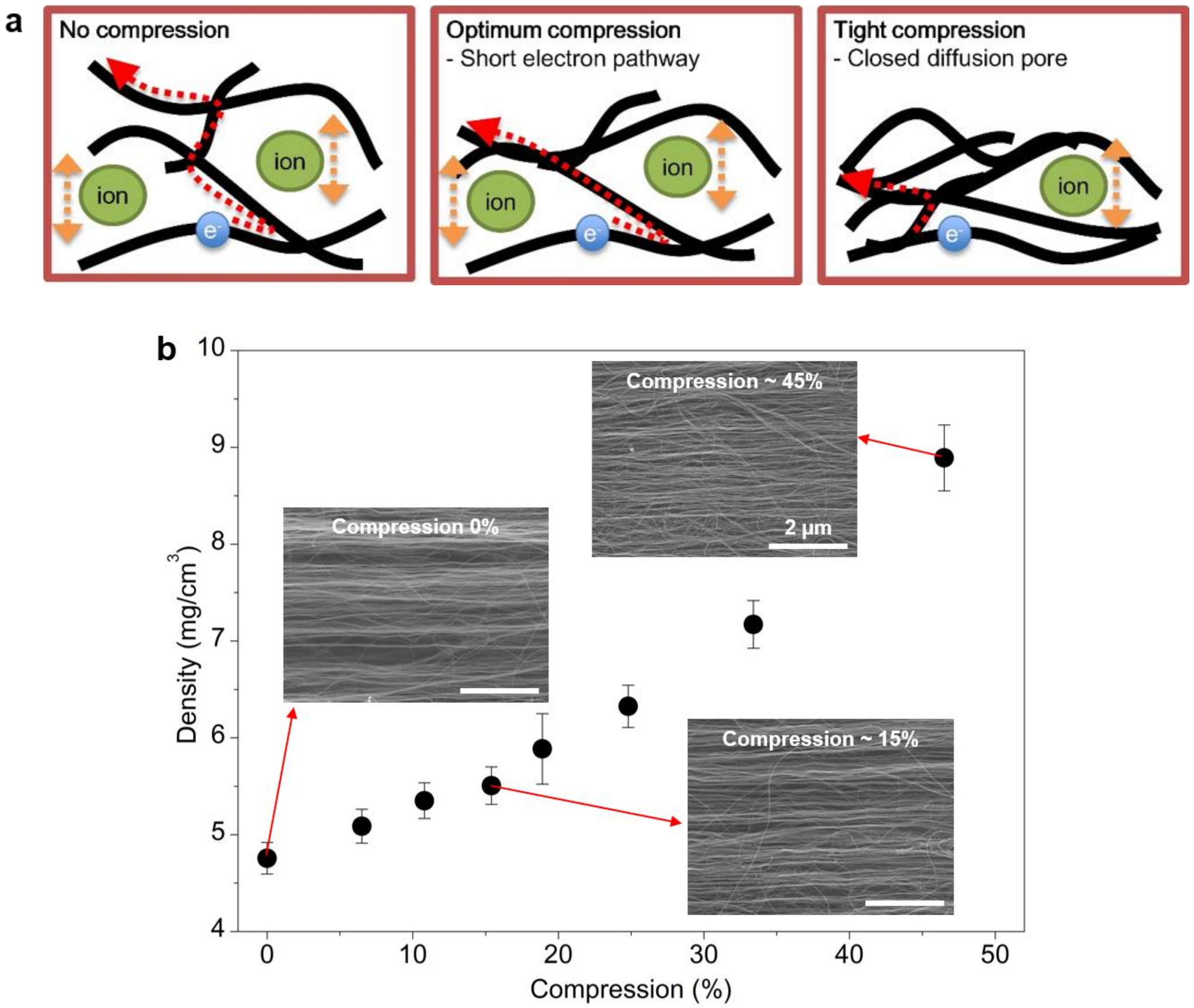

Supplementary Figure 2. Ion transport into the interior of the compressed electrode. (a) Schematic illustration of the effect of mechanical compression on the structure and properties of the CNT aerogel network. The ohmic resistance decreases with increasing compression, but too high a compression decreases pore size, thereby restricting ion diffusion. (b) Density of the CNT aerogel sheet and SEM micrographs (insets) as a function of compression.

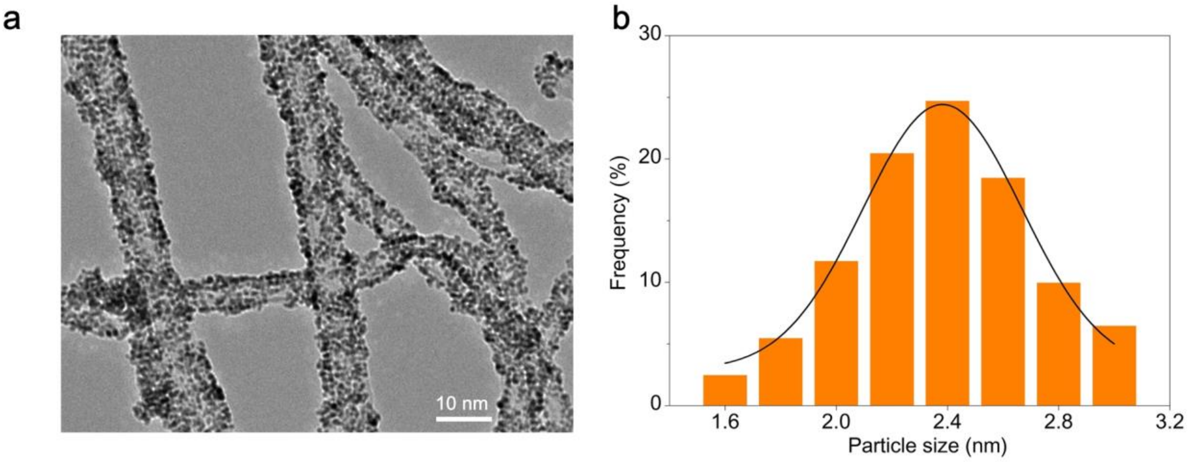

Supplementary Figure 3. Deposition of Pt nanoparticles on the thermally oxidized CNT aerogel sheet electrodes. (a) TEM image of CNTs uniformly decorated with Pt nanoparticles. (b) Size distribution of Pt nanoparticles on CNTs.

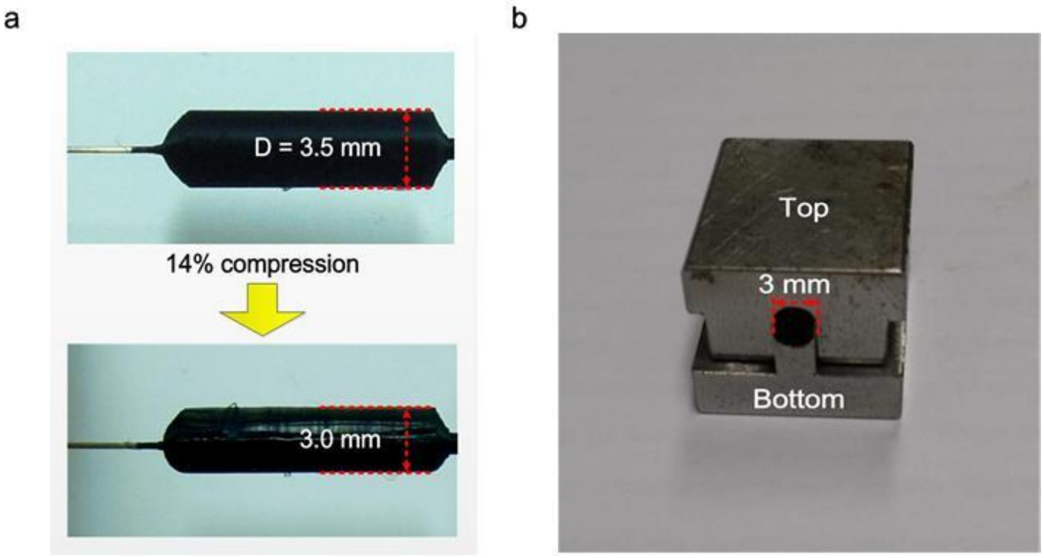

Supplementary Figure 4. Mechanical compression of a CNT sheet electrode. Optical images of (a) cylindrical CNT electrode before (top) and after repeated compressions (bottom) in the compression jig (between which the cylindrical CNT sheet electrode was rotated about its long axis in the compression jig) and (b) the two-part grooved template used as a compression jig for the lateral compression of the cylindrical electrode.

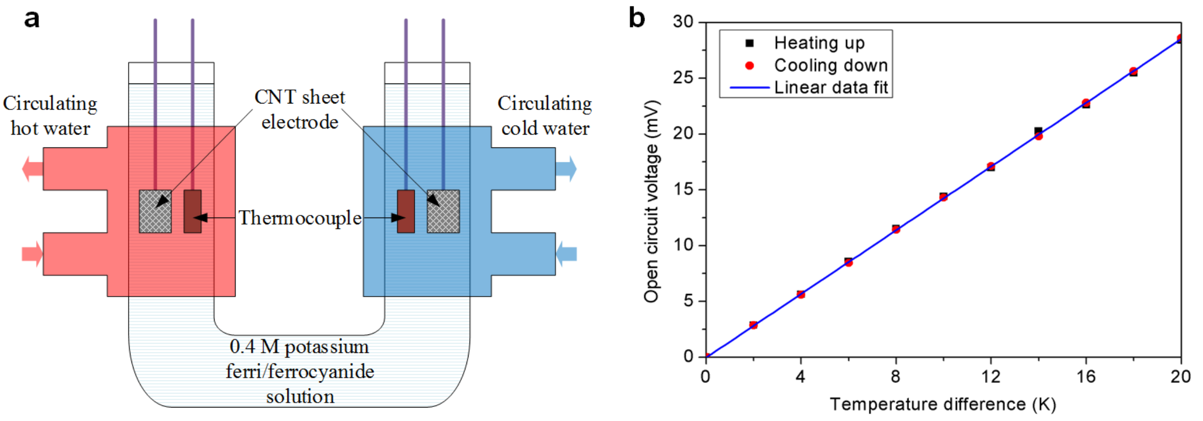

Supplementary Figure 5. Measurement of electrochemical Seebeck coefficient. (a) Schematic drawing of the measurement setup using a U shaped cell. (b) Dependence of open-circuit potential on the temperature difference between the hot and cold electrodes. The electrochemical Seebeck coefficient was measured as ~1.43 mV K^{-1}.

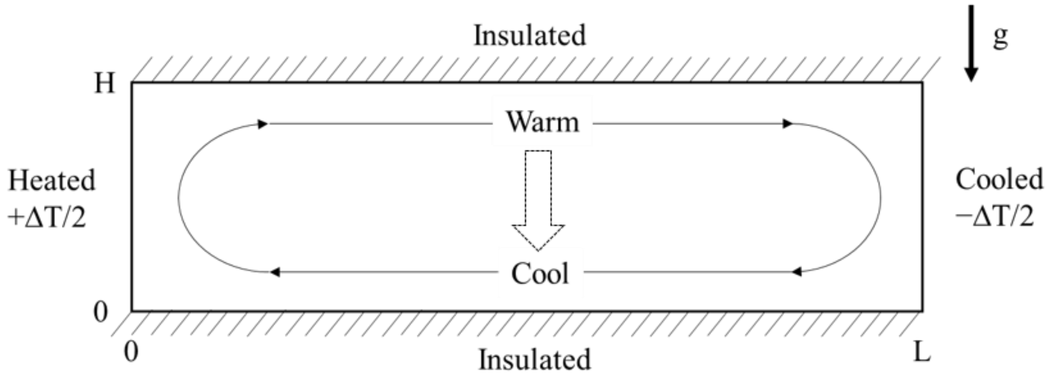

Supplementary Figure 6. Two-dimensional rectangular enclosure between the hot and cold ends with adiabatic sidewalls

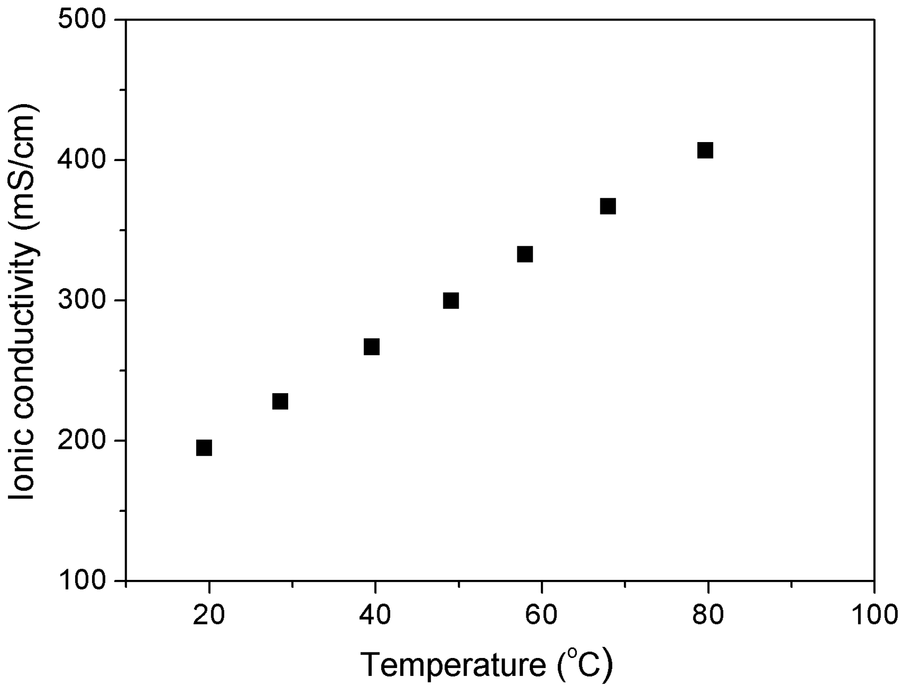

Supplementary Figure 7. Ionic conductivity of a 0.4 M aqueous solution of $Fe(CN)_6^{4-}/Fe(CN)_6^{3-}$ as a function of temperature.

Supplementary Tables

Supplementary Table 1. Physical properties of the electrolyte at 20 °C[1].

Density	1020.5	Kg m^{-3}
Viscosity	1.105×10^{-3}	Kg m^{-1} s^{-1}
Kinematic viscosity	1.083×10^{-6}	m^2 s^{-1}
Diffusivity of $Fe(CN)_6^{3-}$	6.631×10^{-10}	m^2 s^{-1}
Schmidt number	1633	

Supplementary Table 2. Variables and thermal properties of 0.4 M aqueous electrolyte of potassium ferri/ferrocyanide.

Cold electrode temperature (K)	312.3
Hot electrode temperature (K)	363.7
Operating temperature (K)	338
ΔT(K)	51.4
D(m)	0.003
H(m)	0.0015
L(m)	0.025
k (W m^{-1} K^{-1})	0.55
ρ_1 at 312.45K (kg m^{-3})[3]	1090
ρ_2 at 363.85K (kg m^{-3})[3]	1067
α (m^2 s^{-1})	1.8×10^{-7}
ν (m^2 s^{-1})[4]	0.6×10^{-6}
Ra_H	6547.4
$Ra_H^{-1/4}$	0.1112
H/L	0.06

Supplementary Table 3. Parameters used in the conversion efficiency calculation.

Electrochemical Seebeck coefficient (mV K^{-1})	1.43
Effective ionic conductivity (mS cm^{-1})	1210
Operating temperature (K)	338
Hot electrode temperature (K)	363.7
Cold electrode temperature (K)	312.3
Thermal conductivity at the operating temperature (W m^{-1} K^{-1})	0.57
Cross sectional area of the cell (m^2)	7.1×10^{-6}
Inter-electrode spacing (m)	0.025
Internal resistance of the thermocell from a E-I curve shown in Fig. 5d (Ω)	29

Supplementary Notes

Supplementary Note 1: Theoretical mass transfer coefficient for flat electrode plates

Mass transport processes that occur in an electrochemical cell provide three key non-dimensional parameters[1]: the Sherwood number (Sh), the Reynolds number (Re), and the Schmidt number (Sc). These parameters relate the mass transport coefficient with the diffusivity D (m^2 s^{-1}), the characteristic length parameter d_e (m), the fluid velocity u (m s^{-1}) and the kinematic viscosity of the fluid ν (m^2 s^{-1}). The present calculations use the properties of the chosen aqueous electrolyte (5 mM $Fe(CN)_6^{3-}$ and 10 mM $Fe(CN)_6^{4-}$ in 0.5M aqueous NaOH) shown in Supplementary Table 1.

The limiting value of the mass transfer coefficient (k_c), which is theoretical prediction for transport of reactant into a flat plate without hindrance, was calculated as follows from the definition of the Sherwood parameter and the relationship proposed by Leveque for laminar flow in a rectangular channel:

$$\mathrm{Sh} = \frac{k_c \cdot d_\mathrm{e}}{D} = 1.85\left[\mathrm{Re}\cdot\mathrm{Sc}\frac{d_\mathrm{e}}{L}\right]^{1/3}$$

where $\mathrm{Re} = \frac{u \cdot d_e}{\nu}$, $\mathrm{Sc} = \frac{\nu}{D}$, $d_\mathrm{e} = \frac{2(L\times s)}{(L+s)}$, L is the length of the flow channel and s is the inter-electrode distance.

For the used electrolyte flow of 6.6×10^{-6} m^3 s^{-1}, laminar flow of electrolyte is expected in a low Reynolds number regime (Re ~ 650). Using the above equation, the calculated Sh number is ~139, and correspondingly, the theoretically limiting mass transfer coefficient for a flat plate in this electrolyte is predicted to be 5.76×10^{-6} m s^{-1}. While the measured mass transport number of the CNT aerogel electrode (5.19×10^{-6} m s^{-1}) is nearly as high, the measured mass transport number of the CNT buckypaper electrode is much lower (2.51×10^{-6} m s^{-1}).

Supplementary Note 2: Calculation of energy conversion efficiency of thermocells

The energy conversion efficiency (η) of a thermocell is defined as the ratio of maximum electrical power output (P_{max}) from the cell to thermal power flowing through the cell:

$$\eta = \frac{(1/4)V_{OC} \cdot I_{SC}}{A_c \cdot \kappa(\Delta T/d)} \quad (1)$$

where V_{oc} and I_{sc} are the open-circuit voltage and the short-circuit current, respectively, κ is the thermal conductivity of electrolyte, A_c is the cross sectional area of the cell, ΔT is the absolute temperature difference between two electrodes, and d is the inter-electrode spacing.

Applying the relationships, $V_{oc} = \alpha\Delta T$ and $I_{sc} = \alpha\Delta T/R_{cell}$, to Eq. (1), where α is the electrochemical Seebeck coefficient, R_{cell} is the internal resistance of the thermocell, leads to the following equation:

$$\eta = \frac{\alpha^2 \Delta T}{4\kappa} \cdot \frac{d}{A_c \cdot R_{cell}} \quad (2)$$

The theoretical efficiency relative to Carnot efficiency (η_r) can be expressed by the following equation, when Eq. (2) is divided by the Carnot efficiency ($\eta_c = \Delta T/T_H$):

$$\eta_r = \frac{\alpha^2 T_H}{4\kappa} \cdot \frac{d}{A_c \cdot R_{cell}} = \frac{\alpha^2 T_H}{4\kappa} \cdot \sigma_{eff} \quad (3)$$

where σ_{eff} represents the effective conductivity in thermocell, analogous to the electrical conductivity in thermoelectrics. We note that the effective conductivity is evaluated from the internal resistance of the cell.

The resulting calculated Carnot-relative efficiency was η_r = 3.95% for the optimized cylindrical thermocell. The parameters used in the calculation are summarized in Supplementary Table 3.

Supplementary Note 3: Electrochemical Seebeck coefficient and the effective ionic conductivity

A 0.4 M potassium ferri/ferrocyanide aqueous solution with a concentration close to saturation was used as the thermoelectric electrolyte. The electrolyte was prepared using deionized (DI) water and degassed prior to use by bath sonication. The freshly prepared electrolytes were used immediately to avoid the effects of electrolyte degradation. 50 μm thick CNT sheets with identical area of 1.0 cm^2 were used as electrodes. Each CNT sheet electrode was connected to a 0.5 mm diameter platinum (Pt) wire using silver paste which was used to minimize the contact resistance. The contact was then covered by insulating paint to prevent possible artifacts due to interaction between the silver paste and the electrolyte.

A U-shaped cell equipped with liquid flowing pocket at each side was utilized for the measurement of electrochemical Seebeck coefficient (see Supplementary Fig. 5a). The distance between the two half-cells is 3 cm and the temperature of each side cell was controlled by circulating water from a thermostatic bath with an accuracy of ± 0.1 °C. Electrode temperatures were measured using thermocouple probes that were placed in close proximity to the electrode for each half-cell.

The thermoelectric coefficient of the redox couple was obtained by measuring the temperature dependence of the potential difference over a temperature range from 0 to 20 ^{o}C with an increment of ±2 ^{o}C. The potential and current output from the cell was measured using a voltage–current meter (Keithley 2000 multimeter) with 0.002% DC voltage accuracy from 100 nV to 1 KV. As shown in Supplementary Fig. 5b, the thermoelectric coefficient was measured to be ~1.43 mV K^{-1}, which is in good agreement with previous reports.

In thermoelectric devices, an electrical conductivity of the thermoelectric is used to calculate the energy conversion efficiency. It implies that an electrical potential gradient (∇V) is the dominant driving force to transport charges (electrons or holes) in the thermoelectric. However, mass transport (i.e., ion conduction) in thermocells results from both the diffusion processes based on electrical potential gradient (∇V), thermal gradient (Soret diffusion, ∇T), and concentration gradient (Fickian diffusion, ∇c), and the convective process based on density gradient (∇p). In other words, the ion conduction in thermocell is forced by the sum of the above driving forces, not solely by an electrical driving force. Moreover, the discharge behavior of the thermocell is determined by three primary internal resistances (i.e., activation, ohmic and mass transport overpotentials). Therefore, we cannot simply plug an ion conductivity into Eq. (3) but the effective

conductivity (σ_{eff}) should be evaluated from the internal resistance of the cell, i.e., the slope of E–I curve, at a given geometry of thermocell. For instance, the internal resistance of the cylindrical thermocell is measured as ~29 Ω from the E-I curve shown in Fig. 5d. With the cross sectional area of the cell (7.1×10^{-6} m^2) and the inter-electrode spacing (0.025 m), the effective ionic conductivity ($\sigma_{\text{eff}} = \frac{d}{A_{\text{c}} \cdot R_{\text{cell}}}$)) is calculated to be ~1210 mS cm^{-1}.

Supplementary Note 4: Thermal transport in the cylindrical thermocell

Thermal transport in thermocells is generated not only by the heat flow due to thermal conduction through the electrolyte, but also by the additional flow due to all convective processes. Convective heat transfer can be driven by the temperature difference when the electrodes are held in a certain configuration (e.g., the cold above the hot electrode) and by the difference between the densities of the reactants and products in the ongoing reactions at the hot and the cold electrodes. In order to understand how the thermal transport is generated in the present cylindrical thermocell, we conduct a theoretical analysis on the thermal transport as follows:

For an analysis of a quenched convection heat transfer in a horizontally long enclosure between the hot and cold ends, which represents the thermocell of a cylindrical enclosure, consider a two-dimensional enclosure of height *H* and horizontal length *L*, with infinite depth, as shown in Supplementary Fig. 6.

In the internal natural convection, the Rayleigh number based on the enclosure height is defined as

$$\text{Ra}_H = \frac{2\,\text{g}\,(\rho_1 - \rho_2)\,H^3}{\alpha\,\nu\,(\rho_1 + \rho_2)} \qquad (4)$$

Here, g is the gravity, ρ_1 and ρ_2 are the fluid densities near heated and cooled walls, respectively, H is the height of the two-dimensional rectangular enclosure which is configured with two infinite horizontal walls, and α and ν are the thermal diffusivity and kinematic viscosity of fluid, respectively.

For the natural convection condition, the heat transfer rate between the hot and cold end walls is given by[2]:

$$q'_{\substack{\text{convection} \\ \text{left} \rightarrow \text{right}}} \sim k\,\Delta T\,\text{Ra}_H^{1/4} \qquad (5)$$

In the case of shallow enclosure ($\frac{H}{L} \ll 1$), the above convective heat flow from the hot end wall can be diffused vertically downward from the warm upper branch of the circulation flow to the lower branch before reaching the cold end wall (see the dashed arrow in Supplementary Fig. 6). In other words, the convective heat transfer will be quenched down in the middle of the enclosure and diffused back to the hot end wall area. The vertical heat diffusion rate is given by[2]:

$$q'_{\substack{\text{conduction} \\ \text{top} \rightarrow \text{bottom}}} \sim k\, L\, \frac{\Delta T}{H} \qquad (6)$$

If the vertical diffusion rate of Eq. (6) is higher than the convective heat transfer rate of Eq. (5), the energy carried by the upper stream cannot reach the cold end. In this case, the two branches of horizontal counter-flows diffuse to make good thermal contact, which diminishes the convection flow and thus, results in conduction heat transfer dominating across the electrolyte solution, i.e.,

$$k\, L\, \frac{\Delta T}{H} > k\, \Delta T\, \mathrm{Ra}_H^{1/4} \qquad (7a)$$

$$\frac{H}{L} < \mathrm{Ra}_H^{-1/4} \qquad (7b)$$

In applying the above criterion to the present cylindrical enclosure, Eq. (4) should be modified for the cylindrical enclosure using the characteristic length of the infinite horizontal planes with spacing H. The hydraulic diameter equivalence ($D = 4 \times Area/Perimeter$), i.e., the height depicted in Supplementary Fig. 6 corresponds to one half of the diameter of circular cross-section, i.e., $H = 0.5D$. Therefore, Eq. (4) for a cylindrical enclosure featuring a thermo-electrochemical cell of a cylindrical enclosure is given by:

$$\mathrm{Ra}_D = \frac{1/4\, \mathrm{g}\, (\rho_1 - \rho_2)\, D^3}{\alpha\, \nu\, (\rho_1 + \rho_2)} \qquad (8)$$

The variables and thermal properties needed to calculate the Rayleigh number Ra_D for the 0.4M potassium ferri/ferrocyanide as electrolyte are given in Supplementary Table 2.

Here, the density and kinematic viscosity values of the electrolyte are estimated using the temperature-dependent formula given in the literature[3, 4].

Utilizing the values in the table, the reciprocal of quadratic root of Rayleigh number ($\mathrm{Ra}_H^{-1/4}$) is calculated to be 0.11 and the ratio of height to length ($\frac{H}{L}$) is 0.06. These values satisfy Eq. (5) or $\frac{H}{L} < \mathrm{Ra}_H^{-1/4}$, which is the criterion for negligibly small heat transfer to occur because of the aforementioned reasons. This result implies that the convection heat transfer from the heated electrode directing to the cooled electrode is

diffused back to the heated electrode and the thermal conduction should be the dominating mode of the heat transfer through the electrolyte between the hot and cold electrodes.

Supplementary References

1. Wragg, A. A. & Leontaritis, A. A. Local mass transfer and current distribution in baffled and unbaffled parallel plate electrochemical reactors. *Chemical Engineering Journal* 66, 1-10 (1997).

2. Bejan, A. Convection Heat Transfer, 4th ed., pp. 233-241, John Wiley & Sons, Inc., Hoboken, New Jersey (2013).

3. Salazar, P. F., Kumar, S. & Cola, B. A. Design and optimization of thermo-electrochemical cells. *J Appl Electrochem* 44, 325–336 (2014).

4. Romano, M. *et al*. Novel carbon materials for thermal energy harvesting. *J Therm Anal Calorim* 109, 1229-1235 (2012).
